# Effect of weekend versus weekday admission on the mortality of acute ischemic stroke patients in China: an analysis of data from the Chinese acute ischemic stroke treatment outcome registry

**DOI:** 10.3389/fneur.2023.1206846

**Published:** 2023-07-17

**Authors:** Diandian Huang, Yuxuan Lu, Yongan Sun, Wei Sun, Yining Huang, Liwen Tai, Guozhong Li, Huisheng Chen, Guiru Zhang, Lei Zhang, Xuwen Sun, Jinhua Qiu, Yan Wei, Haiqiang Jin

**Affiliations:** ^1^Department of Neurology, Peking University First Hospital, Beijing, China; ^2^Department of Neurology, Second Hospital of Hebei Medical University, Shijiazhuang, China; ^3^Department of Neurology, First Affiliated Hospital of Harbin Medical University, Neurology, Harbin, China; ^4^Department of Neurology, The General Hospital of Shenyang Military Command, Shenyang, China; ^5^Department of Neurology, Penglai People’s Hospital, Penglai, China; ^6^Department of Neurology, Fifth Affiliated Hospital of Sun Yat-sen University, Zhuhai, China; ^7^Department of Neurology, Qindao University Medical College Affiliated Yantai Yuhuangding Hospital, Yantai, China; ^8^Department of Neurology, Huizhou First Hospital, Huizhou, China; ^9^Department of Neurology, Harrison International Peace Hospital, Hengshui, China

**Keywords:** acute ischemic stroke, China, mortality, rural, regional difference, weekend

## Abstract

**Background:**

Due to disparities in medical resources in rural and urban areas as well as in different geographic regions in China, the effect of weekend versus weekday admission on the outcomes of acute ischemic stroke (AIS) patients is unknown. Our aim was to investigate whether the outcomes of AIS patients differ according to the day of admission in China.

**Methods:**

The data were extracted from the Chinese Acute Ischemic Stroke Treatment Outcome Registry (CASTOR), a multicenter prospective study database of patients diagnosed with AIS. The chi-square test (*χ*^2^) and logistic regression were used to assess mortality for weekday and weekend admissions among AIS patients stratified by rural or urban status and geographic region (including the eastern, northeastern, central, and western regions).

**Results:**

In total, 9,256 patients were included in this study. Of these patients, 57.2% were classified as urban, and 42.8% were classified as rural. A total of 6,760 (73%) patients were admitted on weekdays, and 2,496 (27%) were admitted on weekends. There was no significant difference in the mortality rate among patients admitted on weekends compared with those admitted on weekdays in urban (7.5% versus 7.4%) or rural areas (8.8% versus 8.1%; *p* > 0.05). The mortality rate was the highest among patients admitted on weekends and weekdays (11.6% versus 10.3%) in the northeastern area, without statistical significance before and after adjusting for the patients’ background characteristics (*p* > 0.05). In addition, regression analysis revealed that the mortality of patients admitted on weekdays was more likely to be influenced by regional subgroup, hospital level and intravenous thrombolysis than that of patients admitted on weekends.

**Conclusion:**

The weekend effect was not observed in the mortality of patients with AIS regardless of rural–urban status or geographic region in China.

## Introduction

Stroke is the second leading cause of death worldwide (1, 2). The Global Burden of Diseases showed that the annual number of strokes and deaths due to stroke increased substantially from 1990 to 2019 (3). Among the most common types of stroke, acute ischemic stroke (AIS) is the major player (4). Although intravenous thrombolysis and endovascular treatment (EVT) can provide considerable benefits to patients with AIS within a certain time frame (5, 6), stroke ranks as the third leading cause of death in China and is a major component of disability-adjusted life years in China (7).

Stroke mortality is associated with sex and urban and rural disparities in China. According to a previous study, the crude mortality rate for men was higher than that for women. The age-adjusted stroke mortality rates in rural areas are substantially increased compared with those in urban areas (8, 9). The prevalence of stroke was higher in urban areas than rural areas, but the incidence rate and mortality rate of stroke were higher in rural areas than urban areas which illustrated the existence of urban–rural disparity in the burden of stroke in China (10). However, another interesting phenomenon in which the incidence of stroke is increased during off-hour hospital admissions has been dubbed the “weekend effect” (11, 12). A previous study illustrated that this phenomenon was attributed to the decrease in the number of experienced medical service personnel and first-aid provider delays (13), a decline in care quality or a more serious absence of instantaneous neurological deciders (14). Some previous studies have demonstrated the weekend effect, while many studies have reported entirely different results. A study that covered 47,885 patients in Japan found no significant influence on in-hospital mortality by admission day regardless of the initial admission medical ward (15). Another study showed increased mortality owing to weekend emergency admission in Scotland (16). According to the abovementioned reports, it is controversial whether admission to a hospital on a weekday or throughout the weekend leads to a poor prognosis for patients (17).

In China, some studies have explored the weekend effect on acute myocardial infarction (18), subarachnoid hemorrhage (19), and sepsis patients (20). A longer hospital length of stay was observed among stroke patients with weekend admissions in a Taiwanese study (21). A study showed that intracerebral hemorrhage patients admitted during off-hours had a higher risk of poor functional outcomes at 3 months than those admitted during working hours in Chongqing (14). To our knowledge, no study has simultaneously analyzed the weekend effect on mortality among AIS patients by rural and urban differences and regional diversity in stroke care in China. Our study examined the mortality of patients with ischemic stroke independently through data from the Chinese Acute Ischemic Stroke Treatment Outcome Registry (CASTOR) by weekend and weekday admission, rural–urban status and geographic differences to evaluate the influence of the weekend effect throughout China.

## Methods

### Ethics statement

The study protocol was approved by the ethics committee of Peking University First Hospital (IRB approval number: 2015[922]) and all participating hospitals. Written informed consent was obtained from all patients or an appropriate family member (if the patient was unable to provide consent).

### Study design

**Figure 1:**
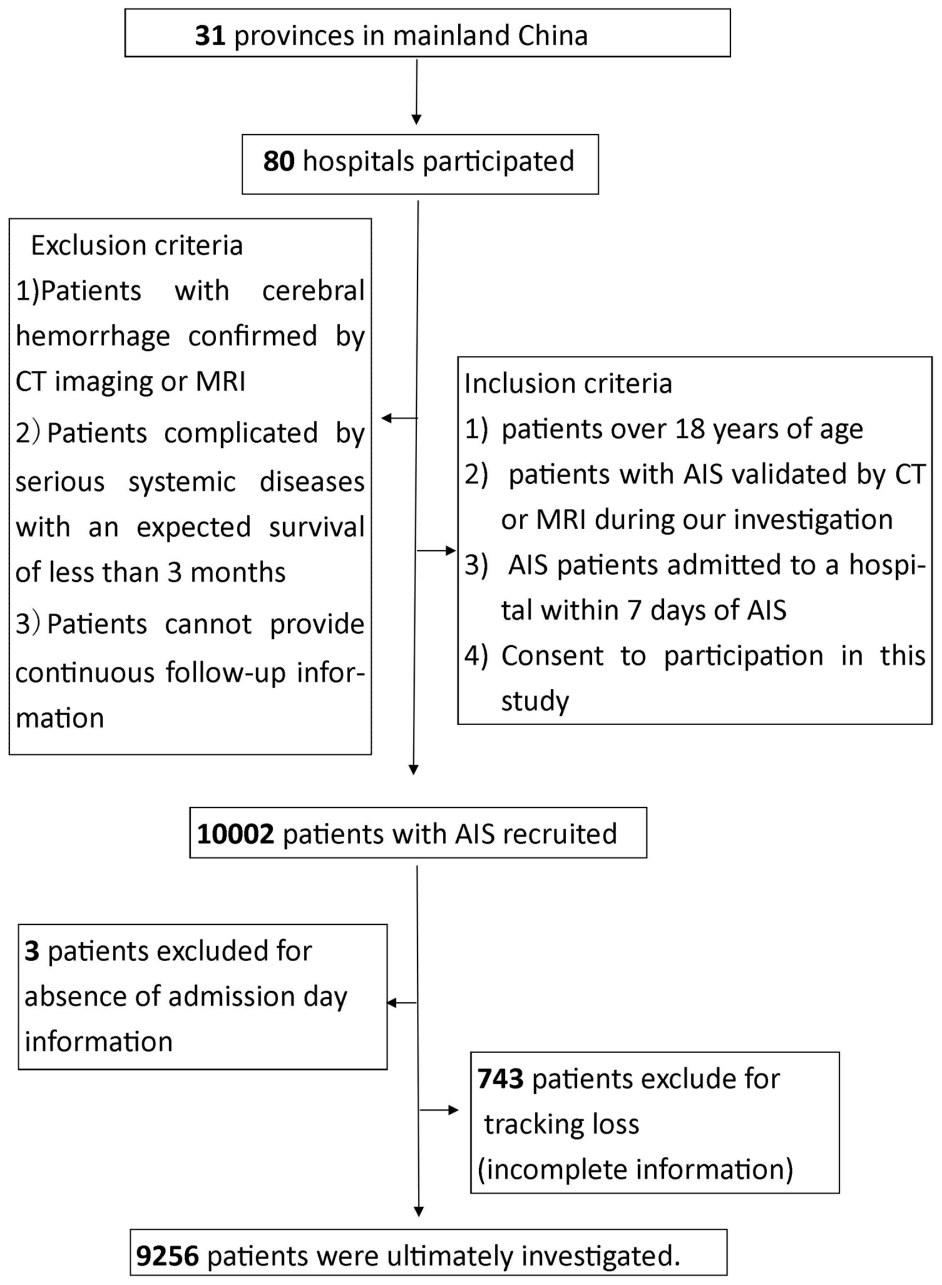
The flow chart in our study.

Data were directly derived from the CASTOR, a multicenter prospective study database. The CASTOR is designed to evaluate the patterns and cost-effectiveness of current treatments for AIS in real-world settings in China (22). A total of 10,002 patients with AIS were recruited in our study; 3 patients were excluded due to the absence of admission day information, 743 patients were excluded because of tracking loss and 9,256 patients were ultimately investigated. The flow chart were added in Figure 1.The experimental project and parameters have been described previously (22). A total of 80 hospitals (including the major tertiary hospitals, sub-tertiary hospitals and the secondary hospitals) participated in our registry, and 35 hospitals (43.75%) had teaching status. It required the National Stroke Center certificate and received guidance from higher-level stroke centers due to the evaluation of the tertiary hospitals required the comprehensive stroke centers (CSC) construction. Secondary hospitals in China were defined as having 100-499 beds, and tertiary hospitals were defined as having more than 500 beds in rural and urban. A total of 5 hospitals were secondary hospitals, and 2 were sub-tertiary hospitals in our study and the rest were major tertiary hospital. A general neurology ward and an intensive care unit (ICU) were evaluated in the investigated hospital. According to the Urban–Rural Classification and Codes (URCC) provided by the China National Bureau of Statistics, which use population and economic development indicators (23), three main urban–rural categories, district, township and countryside, were investigated in our trials. A previous study had the characteristic that cities included only urban areas, excluding counties under the jurisdiction of the cities (24). We defined districts as urban and townships and the countryside as rural.

Therapeutic methods were chosen by the investigators depending on the clinical symptoms, physical signs and medical histories of the patients following the Chinese Guidelines for the Diagnosis and Treatment of Acute Ischemic Stroke (25).

**Figure 2:**
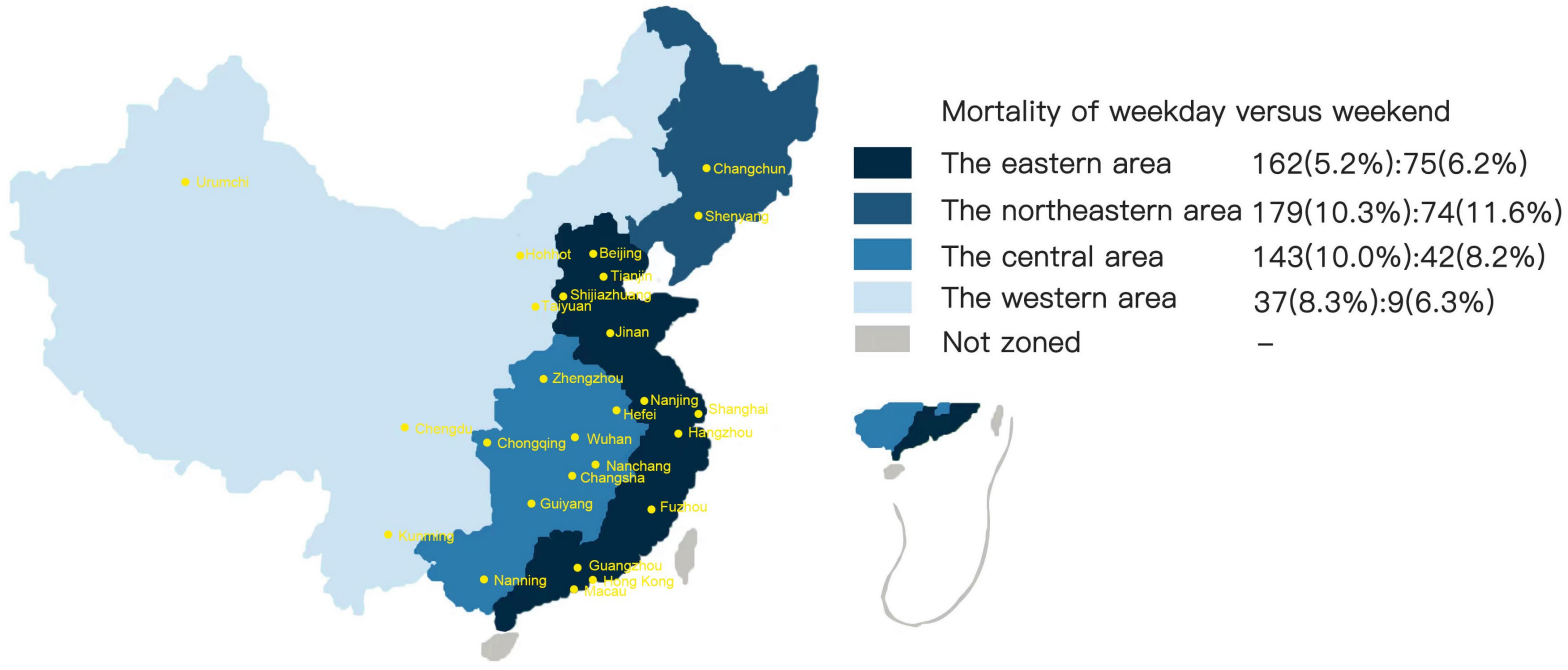
Mortality of acute ischemic stroke patients in different regions of China divided by weekend and weekday admissions.

The main outcome of our study was mortality, a binary variable that was evaluated and defined by the Modified Rankin Scale (mRS) score at discharge and 90 days after the ischemic stroke event. The primary predictor variable was the date of admission. Admission on a Saturday or Sunday was coded as 1, and admission from Monday to Friday was coded as 0. The secondary predictor variable included rural–urban location and regional diversity among AIS patients. Regional distribution was divided into four groups: the eastern region, northeastern region, central region and western region (26) (Figure 2).

### Explanatory variables

The confounding variables were sex, age, payment type, in-hospital complications, hospital level, ICU admission, National Institutes of Health Stroke Scale (NIHSS) score on admission, Charlson Comorbidity Index score and thrombolysis.

The age groups were classified as 18–44 years, 45–64 years, 65–74 years, and 75 years and older. Payment type was mainly separated into two groups: the self-pay group and the medical insurance group. In-hospital complications included infection, deep venous thrombosis, cardiac events and hemorrhage. The designation of in-hospital complications was identical to that in previous studies (27). Infection mainly included pulmonary infection, urinary infection and so on. Hemorrhage primarily occurred in the gastrointestinal tract and urinary and integumentary systems, and hemorrhagic stroke stemmed from ischemic stroke (28). Hospital level was separated into tertiary hospitals and secondary hospitals. Tertiary hospitals (with more than 501 beds) were defined as regional and advanced medical service centers that provide comprehensive medical services for patients and perform advanced education and scientific research tasks within the region and surrounding areas. Secondary hospitals (with more than 100 beds) were defined as regional hospitals that are county centered, provide urgent and preventative medical services to multiple communities for over 10,0000 patients and perform certain teaching and research tasks (26). NIHSS scores were ascertained on the date of admission, and patients were divided into mild (0–5), moderate (6–10) and severe (> 10) groups (29). The Charlson Comorbidity Index was the primary evaluation method for the number of comorbidities, and patients were divided into two groups: a group with 1 comorbidity and a group with 2 or more comorbidities. Comorbidities in our study were classified as prior stroke, hypertension, coronary heart disease (CHD), diabetes mellitus (DM), and atrial fibrillation (AF). Prior stroke included ischemic stroke and hemorrhagic stroke. Hypertension was defined as a diagnostic history of increased arterial blood pressure (systolic blood pressure ≥ 140 mmHg, diastolic blood pressure ≥ 90 mmHg) on two subsequent occasions or the use of antihypertensive drugs. DM was defined as a history of increased blood glucose levels on two substantive occasions or the use of drugs for DM. CHD was classified as any previous heart attack and/or myocardial infarction, angina, or coronary heart disease. AF was defined as previous AF or AF determined with ECG during the hospital stay. In the CASTOR investigation, the mRS scores were evaluated in the admission day, the discharge day, and the 90 days and the 360 day of occurrence, respectively. We use the mRS score for binary selection, selection only included the death and alive.

### Statistical analysis

Statistical analysis was performed by evaluating the complete data and excluding patients with missing data (9,256 patients were ultimately enrolled). ICD-10 codes and the HCUPNet recommended coding instructions^1^ were used to evaluate stroke patients independently, and the two methods were used equally.

First, we investigated the background characteristics of the patients categorized by the day of admission and AIS mortality by personal-and hospital-level factors using Pearson’s Chi-square test (*χ*^2^).

Then, logistic regression was conducted for mortality between AIS patients with weekend and weekday admissions, modified by sex, age, payment type, medical history, location (urban and rural), in-hospital complications, geographic region, hospital level, ICU admission, NIHSS score at admission and thrombolysis. These confounding variables were selected based on a previous study that illustrated differences in mortality by patient variance and distinct Chinese hospital characteristics (29, 30). SPSS (version 27.0) software was used for statistical analysis.

## Results

**Figure 3:**
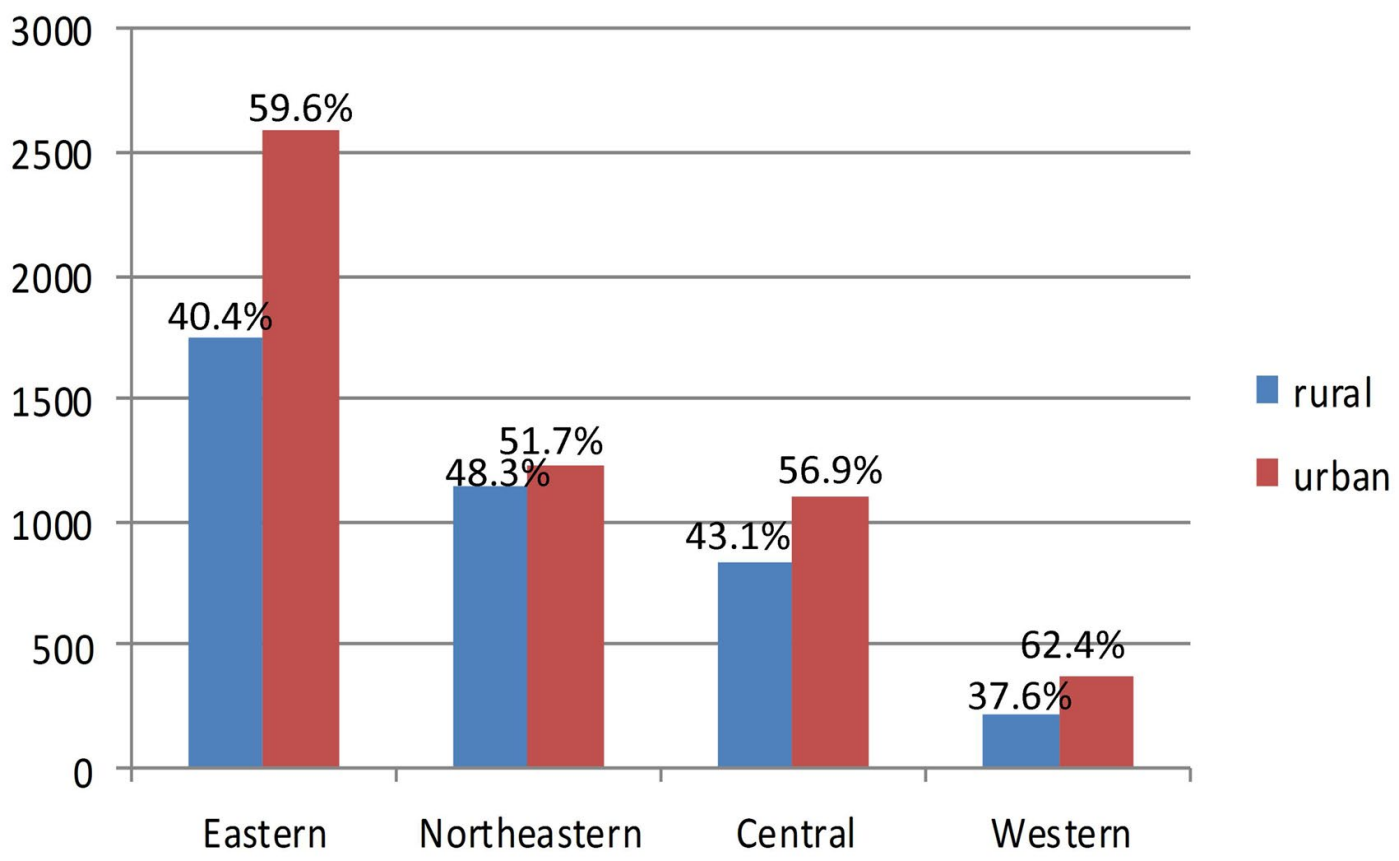
Comparison between rural and urban acute ischemic stroke (AIS) patients in the regional groups.

9,256 patients were included in our study ultimately. For the CASTOR results, in the rural AIS patients, 90.2% were admitted to major tertiary hospitals (*n* = 4,771), 2.2% were admitted to sub-tertiary hospitals (*n* = 119) and 7.6% were admitted to secondary hospitals (*n* = 402). In the urban AIS patients, 90.0% were admitted to major tertiary hospitals (*n* = 3,569), 1.8% were admitted to sub-tertiary hospitals (*n* = 70) and 8.2% were admitted to secondary hospitals (*n* = 325). The percentages of AIS patients living in rural and urban areas and in separated geographic regions are shown in Figure 3. The mortality rate in the mild group was 6.5% (*n* = 5,882), the mortality rate in the moderate group was 7.4% (*n* = 2,112), and the mortality rate in the severe group was 14.3% (*n* = 1,259).

The background characteristics of AIS patients by weekday versus weekend admission and mortality are presented in Table 1. Approximately 60% of all admissions and deaths occurred among males. Over 65% of the patients were male, and most patients were aged 45–64 years, accounting for 40.6% of deaths. Payment type did not show a discrepancy in mortality between patients with weekday admissions and those with weekend admissions. More than 90% of patients were admitted to a major tertiary hospital. ICU admissions were increased on weekends (5.7%) compared with weekdays (3.3%), which was statistically significant (*p* < 0.05). NIHSS scores showed that over 60% of the participants had a mild case of stroke. There was no obvious statistically significant difference in the number of patients who received thrombolysis therapy between patients admitted on weekends and weekdays (*p* > 0.05). In addition, there was no statistical significance between weekday and weekend admission AIS patients who underwent thrombectomy in our study.

**Table 1:** Patient characteristics by weekday versus weekend admission and stroke mortality among patients with ischemic stroke in the CASTOR database (*n* = 9,256).

Variable	Weekday	Weekend	*p* Value	No mortality	Mortality	*p* Value	Mortality
*Sex*			0.824			<0.001	
Male	4,453 (65.9%)	1,638 (65.6%)		5,660 (66.3%)	431 (59.8%)		
Female	2,307 (34.1%)	858 (34.4%)		2,875 (33.7%)	290 (40.2%)		
*Age group*			0.297			<0.001	
18–44	334 (4.9%)	145 (5.8%)		445 (5.2%)	34 (4.7%)		
45–64	3,160 (46.7%)	1,139 (45.6%)		4,045 (47.4%)	254 (35.2%)		
65–74	1866 (27.6%)	707 (28.3%)		2,376 (27.8%)	197 (27.3%)		
75+	1,400 (20.7%)	505 (20.2%)		1,669 (19.6%)	236 (32.7%)		
*Payment type*			0.073			0.319	
Self-paying	1,102 (16.3%)	446 (17.9%)		1,437 (16.8%)	111 (15.4%)		
Medical insurance	5,658 (83.7%)	2050 (82.1%)		7,098 (83.2%)	610 (84.6%)		
*In-hospital complications*							
Deep venous thrombosis	6 (0.01%)	3 (0.01%)	0.667	8 (0.01%)	1 (0.01%)	0.710	
Hemorrhage	128 (1.9%)	52 (2.1%)	0.886	145 (1.7%)	35 (4.9%)	<0.001	
Cardiac events	20 (0.3%)	9 (0.3%)	0.883	19 (0.2%)	10 (0.4%)	<0.001	
Infection	459 (6.8%)	181 (7.3%)	0.519	538 (6.3%)	102 (14.1%)	<0.001	
*Hospital level*			0.835			<0.001	
Major tertiary hospital	6,095 (90.2%)	2,245 (89.9%)		7,673 (89.9%)	667 (92.5%)		
Sub-tertiary hospital	140 (2.1%)	49 (2.0%)		168 (2.0%)	21 (2.9%)		
Secondary hospital	525 (7.8%)	202 (8.1%)		694 (8.1%)	33 (4.6%)		
*ICU admission*	232 (3.4%)	93 (3.7%)	<0.001	284 (3.3%)	41 (5.7%)	0.004	
*NIHSS score on admission*			0.323			<0.001	
Mild (0–5)	4,319 (63.9%)	1,564 (62.7%)		5,500 (64.4%)	383 (53.1%)		
Moderate (6–10)	1,517 (22.4%)	597 (23.9%)		1957 (22.9%)	157 (21.8%)		
Severe (>10)	924 (13.7%)	335 (13.4%)		1,078 (12.6%)	181 (25.1%)		
*Comorbidities*			0.372			<0.001	
0–1	3,917 (57.9%)	1,472 (59.0%)		5,054 (59.2%)	335 (46.5%)		
2	2,843 (42.1%)	1,024 (41.0%)		3,481 (40.8%)	386 (53.5%)		
*Thrombolysis*	295 (4.4%)	112 (4.5%)	0.797	381 (4.5%)	26 (3.6%)	0.281	
*Geographic region*			0.264			<0.001	
Eastern	3,140 (46.4%)	1,204 (48.2%)		4,107 (48.1%)	237 (32.9)		5.5%
Northeastern	1742 (25.8%)	638 (25.6%)		2,127 (24.9%)	253 (35.1)		10.6%
Central	1,430 (21.2%)	511 (20.5%)		1756 (20.6%)	185 (25.7)		9.5%
Western	448 (6.6%)	143 (5.7%)		545 (6.4%)	46 (6.4%)		7.8%
*Location*			0.268			0.153	
Urban	3,856 (57.0%)	1,436 (57.6%)		4,898 (57.4%)	394 (54.6%)		7.4%
Rural *Thrombectomy*	2,906 (43.0%) 16 (0.2%)	1,058 (42.4%) 6 (0.2%)	0.988	3,637 (42.6%) 21 (0.2%)	327 (45.4%) 1 (0.1%)	0.849	8.1%

NIHSS: National Institutes of Health Stroke Scale. ICU: intensive care unit.

The *χ*^2^ tests for subgroup analyses of the weekend effect revealed no difference between patients from urban and rural areas (Table 2) or in regional variance (Table 3, Figure 2). After adjusting for confounding variables, the difference in mortality by urban–rural status or regional subgroup was not statistically significant. Table 3 also shows that the northeastern region had the highest mortality on weekdays (10.3%) and weekends (11.6%) compared to other regions.

**Table 2:** Mortality between weekday and weekend admissions among ischemic stroke patients by rural–urban status.

		Weekday^a^	Weekend^a^	*p* Value	OR	95% CI
Rural mortality	Unadjusted	235 (8.1%)	92 (8.8%)	0.488	–	–
	Adjusted^b^	–	–	0.702	0.951	0.733–1.233
Urban mortality	Unadjusted	286 (7.4%)	108 (7.5%)	0.961	–	–
	Adjusted^b^	–	–	0.811	0.972	0.770–1.229
*p* Value		0.334	0.245			

*p* Values were calculated with Chi-square tests.

a The results are presented as *N* (%).

b Models were adjusted for age, sex, payment type, in-hospital complications, hospital level, ICU admission, Charlson Comorbidity Index score, NIHSS score on admission, and thrombolysis. Marginal probabilities were estimated from the logistic regression model and converted from odds ratios.

**Table 3:** Analysis of regional differences and mortality between weekday and weekend admissions.

		Weekday admissions^a^	Weekend admissions^a^	*p* Value	OR	95% CI
Eastern region mortality	Unadjusted	162 (5.2%)	75 (6.2%)	0.165	–	–
	Adjusted^b^			0.116	0.795	0.598–1.059
Northeastern region mortality	Unadjusted	179 (10.3%)	74 (11.6%)	0.354	–	–
	Adjusted^b^	–	–	0.469	0.897	0.667–1.205
Central region mortality	Unadjusted	143 (10.0%)	42 (8.2%)	0.239	–	–
	Adjusted^b^	–	–	0.213	1.266	0.873–1.836
Western region mortality	Unadjusted	37 (8.3%)	9 (6.3%)	0.445	–	–
	Adjusted^b^	–	–	0.529	1.284	0.591–2.790

a The results are presented as *N* (%).

b Models were adjusted for age, sex, payment type, in-hospital complications, hospital level, ICU admission, Charlson Comorbidity Index score, NIHSS score on admission, and thrombolysis. Marginal probabilities were estimated from the logistic regression model and converted from odds ratios.

Overall, logistic regression for the confounding variables, including the hospital level, thrombolysis and regional subgroups, suggested significant differences (*p* < 0.05) in the analyses of mortality on weekdays compared with weekends (Table 4).

**Table 4:** Logistic regression analysis of mortality between weekday and weekend admissions.

	Weekday			Weekend		
	*p* Value	OR	Coefficient (95% CI)	*p* Value	OR	Coefficient (95% CI)
Age	0.000	1.283	1.151–1.430	0.007	1.278	1.070–1.526
Sex	0.357	1.094	0.904–1.323	0.238	1.203	0.885–1.635
Payment type	0.428	0.905	0.708–1.158	0.063	1.532	0.977–2.403
In-hospital complications	0.000	2.107	1.636–2.713	0.000	2.122	1.429–3.151
Hospital level	0.045	0.803	0.648–0.996	0.610	0.928	0.697–1.236
ICU admission	0.096	1.438	0.938–2.205	0.460	1.292	0.655–2.547
NIHSS score on admission	0.000	1.325	1.179–1.490	0.000	1.453	1.204–1.755
Charlson Comorbidity Index score	0.000	1.446	1.202–1.738	0.002	1.626	1.203–2.197
Thrombolysis	0.030	0.560	0.332–0.946	0.616	0.828	0.397–1.728
Regional subgroup	0.000	1.267	1.156–1.390	0.292	1.089	0.929–1.277
Location	0.173	1.136	0.945–1.366	0.312	1.168	0.865–1.578

## Discussion

The overall mortality rate in our study was 7.8%, with rates of 7.4% for urban patients and 8.2% for rural patients with AIS. The mortality rate among AIS patients in our study was similar to that in a study from Japan (15) but higher than that in a study from the United States (31). The mortality rate of the group with a mild NIHSS score was 6.3%, which was in line with the range of the previous study, which ranged from 9.4% (mechanical thrombectomy group) to 3.5% (best medical management group) (32). There was no rural–urban difference in the mortality rate between patients admitted on weekdays and weekends in our study; this was contrary to the urban–rural bias in China reported in another study, which disregarded the date difference (33). A Korean study investigated 8,957 AIS patients and reported that the weekend effect on ischemic stroke patients was associated with increased in-hospital and 30-day mortality rates, while the 7-day mortality rate showed no statistical significance, which was partially in accordance with our study (34). In China, Ding et al. stated that off-hours admission was related to latency in the reperfusion time of EVT among AIS patients, but the delay was not associated with deteriorated functional appearance or higher mortality (35). However, their study included only some adult patients with acute large vessel occlusion who received EVT, which did not cover all AIS patients. Regarding regional subgroups, the mortality rate associated with weekday and weekend admissions showed no statistically significant difference (*p* > 0.05), even though the northeastern area had a higher mortality rate than other geographical areas.

Patient clinical characteristics and backgrounds at admission were practically consistent regardless of admission day, but ICU admission significantly differed between weekdays and weekends. A Japanese study demonstrated a tendency of delaying admission of nonfatal patients from the weekend to a weekday (36). Patients experiencing disease on the weekend are regularly admitted to an ICU due to ICU hospital staffing on these days (15). In addition to ICU admission, the majority of our patients’ characteristics were similar to those of patients in multiple formal studies (37–39).

Multiple studies on the off-hours admissions of patients with cerebrovascular and cardiovascular events have explained the weekend effect (35, 40–44). In a study involving 8 CSCs in the Specialized Programs of Translational Research in Acute Stroke (SPOTRIAS) cohort, the weekend effect was not observed (42). Zha et al. (45) indicated that the off-hours effect could be eliminated by considering clinical consequences and in-hospital workflows in anterior circulation large vessel occlusion patients receiving EVT. Profiting from 7/24 capabilities of stroke expertise, proficient stroke nursing specialists, and neuroimaging abilities, CSCs might eliminate the previously reported weekend effect among AIS patients (37). In addition, neurointerventional groups, which involve neurologists, nurses and cerebrovascular interventional radiologists, could perform multiplex treatment, such as intravenous thrombolysis and EVT (46). These studies elucidated how differences between weekday and weekend admission among stroke patients could be eliminated over time and with different developed treatment methods.

Based on 2016 National Inpatient Sample data, a United States study did not observe a relationship between mortality and admission day among AIS patients with rural–urban diversity, which was attributed to updated investigations and the efforts of modern stroke care quality promotion (31). A study reported that rural AIS patients treated in hospitals in agricultural regions had similar benefits and safety from thrombolytic treatment in Portland and even had a lower chance of intravenous thrombolysis at night, and improvements in telecommunication and patient transport from rural areas during the night weakened the weekend effect (47). A recent study showed that there was no difference in intravenous recombinant tissue plasminogen activator (IV-tPA) treatment times, acute stroke measurement times, or mortality between patients who received delayed or on-time treatment. All-hours telecommunication with stroke experts can ensure appropriate treatment and adequate care for AIS patients regardless of the admission time or day, which could reduce the weekend effect in urban and rural and geographically different areas (48). In China, telemedicine application was shown to accelerate the bed occupancy rate of township health centers in the national range. Furthermore, telemedicine was found to improve the number of annual outpatient visits in Western China and the bed occupancy rate in eastern China, which may contribute to the elimination of the weekend effect in rural and urban areas (49). In addition, the narrowing of the gap between urban and rural mortality might be related to improved health care in rural regions after several poverty alleviation campaigns and increased rural medical insurance coverage after the implementation of the New Rural Cooperative Medical Scheme in 2003 (33). In the stable domestic environment, the increase in public revenue and the updating of the public health system promoted county CSC construction and the training of stroke specialists. Besides, about 90% of admissions were in tertiary centers, but about 57% of patients were in urban locations. On one side, due to the considerable gaps in healthcare resources and medical techniques between tertiary hospitals and primary health centers and patients’ distrust of primary health centers, the majority of Chinese patients prefer to choose tertiary hospitals after the occurrence of disease (50). On the other side, with the improvement of infrastructure in rural areas, the cost of time from rural areas to urban areas is also shortening. Besides, our definition of the urban and rural was in accordance with the living status which may affect by the urbanization process (51) and the rural–urban immigrations (52). Due to the aforementioned methods, the definition of the rural and the urban may be weakened.

Regarding the prediction of mortality on weekdays and weekends, following a previous study, age (53), Charlson Comorbidity Index score (54), NIHSS score (54), and in-hospital complications (55) were the apparent predictors for both weekday and weekend admissions. Our study also indicated that intravenous thrombolysis, hospital level and regional differences may affect mortality on weekdays. Compared with no reperfusion therapy, IV-tPA has been shown to improve functional outcomes after AIS in randomized trials (5, 56). The accurate factors for the conflicting outcomes of the weekend effect among AIS patients are not entirely understood. The reported IVT rates on weekends have been rather higher than (57, 58) or equal (59) to weekdays and do not therefore provide an explanation. In addition, the concept of the weekend effect has recently been queried with a more sophisticated correlation of admission time and outcomes (60). Hospital-level outcomes varied considerably for AIS patients in America in terms of mortality and rehospitalization (61). In addition, stroke occurrence, mortality, and prevalence fluctuate extensively among various regions within China, with a conspicuous North–South gradient (62), which may compromise the variation in the regression for our study.

## Limitations

This study had several limitations. First, due to the retrospective nature of this study, selection bias may have occurred, as the day of stroke occurrence was uncontrollable, which may have influenced the treatment strategy. Second, the regional data were not sufficient, and most of the AIS patients were admitted to first-class hospitals. Future studies require more comprehensive data coverage and analysis. Third, the mortality and demographic data came from manual medical records, and recording errors are probable. Fourth, the possibility of unrecorded comorbid conditions and other interrelated confounders cannot be excluded. Finally, our definition of urban and rural was in accordance with living status, which may be affected by the urbanization process and rural–urban immigrations.

## Conclusion

In summary, our study did not show any weekend effect on the mortality of AIS patients after adjusting for the patients’ background characteristics, regardless of location and regional differences.

## Data availability statement

The raw data supporting the conclusions of this article will be made available by the authors, without undue reservation.

## Ethics statement

The studies involving human participants were reviewed and approved by the ethics committee of Peking University First Hospital (IRB approval number: 2015[922]) and all participating hospitals. Written informed consent was obtained from all the patients or an appropriate family member (if the patient was unable to provide it) to participate. The patients/participants provided their written informed consent to participate in this study.

## Author contributions

HJ and YH: conceptualization. DH and YL: data curation. YS, WS, LT, GL, HC, GZ, LZ, XS, and JQ: investigation. DH: methodology and writing—original draft. YH: supervision. JH: writing—review and editing. All authors contributed to the article and approved the submitted version.

## Funding

This work was supported by the National Natural Science Foundation of China (No. 82071306). The CASTOR study was funded by Techpool Bio-Pharma Co., Ltd. All funders were not involved in the study design, collection, analysis, interpretation of data, the writing of this article, or the decision to submit it for publication. All authors declare no other competing interests.

## Footnotes

^1^ https://hcupnet.ahrq.gov/#setup
